# Effects of a Chatbot-Based Intervention on Stress and Health-Related Parameters in a Stressed Sample: Randomized Controlled Trial

**DOI:** 10.2196/50454

**Published:** 2024-05-28

**Authors:** Christine Schillings, Echo Meißner, Benjamin Erb, Eileen Bendig, Dana Schultchen, Olga Pollatos

**Affiliations:** 1 Department of Clinical and Health Psychology Institute of Psychology and Education Ulm University Ulm Germany; 2 Institute of Distributed Systems Ulm University Ulm Germany; 3 Department of Clinical Psychology and Psychotherapy Institute of Psychology and Education Ulm University Ulm Germany

**Keywords:** chatbot, intervention, stress, interoception, interoceptive sensibility, mindfulness, emotion regulation, RCT, randomized controlled trial

## Abstract

**Background:**

Stress levels and the prevalence of mental disorders in the general population have been rising in recent years. Chatbot-based interventions represent novel and promising digital approaches to improve health-related parameters. However, there is a lack of research on chatbot-based interventions in the area of mental health.

**Objective:**

The aim of this study was to investigate the effects of a 3-week chatbot-based intervention guided by the chatbot ELME, specifically with respect to the ability to reduce stress and improve various health-related parameters in a stressed sample.

**Methods:**

In this multicenter two-armed randomized controlled trial, 118 individuals with medium to high stress levels were randomized to the intervention group (n=59) or the treatment-as-usual control group (n=59). The ELME chatbot guided participants of the intervention group through 3 weeks of training based on the topics stress, mindfulness, and interoception, with practical and psychoeducative elements delivered in two daily interactive intervention sessions via a smartphone (approximately 10-20 minutes each). The primary outcome (perceived stress) and secondary outcomes (mindfulness; interoception or interoceptive sensibility; subjective well-being; and emotion regulation, including the subfacets reappraisal and suppression) were assessed preintervention (T1), post intervention (T2; after 3 weeks), and at follow-up (T3; after 6 weeks). During both conditions, participants also underwent ecological momentary assessments of stress and interoceptive sensibility.

**Results:**

There were no significant changes in perceived stress (β_03_=–.018, SE=.329; *P*=.96) and momentary stress. Mindfulness and the subfacet reappraisal significantly increased in the intervention group over time, whereas there was no change in the subfacet suppression. Well-being and momentary interoceptive sensibility increased in both groups over time.

**Conclusions:**

To gain insight into how the intervention can be improved to achieve its full potential for stress reduction, besides a longer intervention duration, specific sample subgroups should be considered. The chatbot-based intervention seems to have the potential to improve mindfulness and emotion regulation in a stressed sample. Future chatbot-based studies and interventions in health care should be designed based on the latest findings on the efficacy of rule-based and artificial intelligence–based chatbots.

**Trial Registration:**

German Clinical Trials Register DRKS00027560; https://drks.de/search/en/trial/DRKS00027560

**International Registered Report Identifier (IRRID):**

RR2-doi.org/10.3389/fdgth.2023.1046202

## Introduction

Stress levels and the prevalence of mental disorders in the general population have been rising in recent years, which have been further accelerated by the COVID-19 pandemic [1]. Digital mindfulness-based interventions were indicated as promising approaches to improve mental health outcomes such as stress (eg, [2-4]), mindfulness (eg, [2,3]), or subjective well-being (eg, [3]), highlighting the crucial role of emotion regulation [5]. In particular, guided online interventions are of high relevance, as they are associated with higher adherence rates than unguided interventions [6-9]. Novel digital approaches of increasing interest include support from chatbots [10-12], which can be used anonymously, regardless of time and location, and are easily integrated into individuals’ everyday lives [6,13-16].

Studies of chatbot-based interventions aiming to improve mental health outcomes have provided evidence for decreases in distress [17-20] or increased subjective and psychological well-being [18,21,22]. Importantly, randomized controlled trials (RCTs) in the context of mental health are sparse and inconsistent [12,20,23-25] and there is a lack of research on the efficacy of chatbot-based interventions [20,23,24], especially for emotion regulation and interoception [26,27]. Given the impairment of interoceptive abilities in mental disorders (eg, [28,29]) or under long-term stress [30], approaches that can help to train interoceptive abilities are essential, which can be achieved via mindfulness-based interventions (eg, [31,32]).

Overall, there is a need for research on chatbot-based interventions considering standardized characteristics (eg, intervention duration, samples, outcome assessments) and guidelines. Furthermore, interoception has not been the focus of previous research on chatbot-based interventions, neither being included as part of the intervention contents nor implemented as ecological momentary assessment (EMA) measures. EMA represents a flexible approach to measure real-time data, including health data, in everyday life [33]. Therefore, to fill these gaps, we developed a new chatbot-based intervention fostering the abilities of interoception, mindfulness, and stress management in everyday life.

The aim of this study was to investigate the effects of a 3-week chatbot-based intervention on stress, mindfulness, interoception, subjective well-being, and emotion regulation in individuals with medium to high stress levels. Based on previous findings, perceived stress was chosen as the primary outcome. Further details are described in the study protocol [34].

We hypothesized that: (1) the primary outcome (perceived stress) will be reduced in the intervention group compared to the treatment-as-usual control group over time, as assessed at preintervention (T1), post intervention (T2), and at the 3-week follow-up (T3) and via EMA; and (2) the secondary outcomes (mindfulness; interoception, including interoceptive sensibility; subjective well-being, and emotion regulation) will be improved in the intervention group compared to the control group, as assessed at T1, T2, and T3. Momentary interoceptive sensibility and stress were also assessed via EMA.

Furthermore, adherence, dropout reasons, usability, and user feedback regarding the intervention were assessed to potentially further improve the intervention for future research.

## Methods

### Setting and Recruitment

The data collection took place between February and September 2022. German-speaking people were recruited via offline and online recruitment strategies. Participants were included in the study if they (1) were 18 years or older, (2) had sufficient knowledge of the German language, (3) owned a smartphone (Android or iOS) with internet access, (4) possessed a valid smartphone number, (5) possessed a valid mailing address, (6) experienced a middle to high level of perceived stress (according to a 10-item Perceived Stress Scale [PSS-10] score≥14, assessed at screening [T0]), (7) were not diagnosed with any mental disorder, (8) did not undertake psychotherapy, and (9) were not currently participating in another online mental health intervention.

### Study Design

The intervention group received a 3-week online-based intervention guided by the chatbot ELME. The control group received treatment as usual (ie, no content and only answered the questionnaires and the EMAs). Primary and secondary outcomes were assessed in both groups at T0, T1, daily during the intervention (between T1 and T2), T2, and T3. The design of the study and the usability of the chatbot were successfully tested in a previous feasibility study [35]. The trial was registered a priori at the World Health Organization (WHO) International Clinical Trials Registry Platform via the German Clinical Studies Trial Register (DRKS00027560) on January 6, 2022. The detailed design of this two-armed, parallel RCT is presented in the published study protocol [34].

### Study Procedure

**Figure 1:**
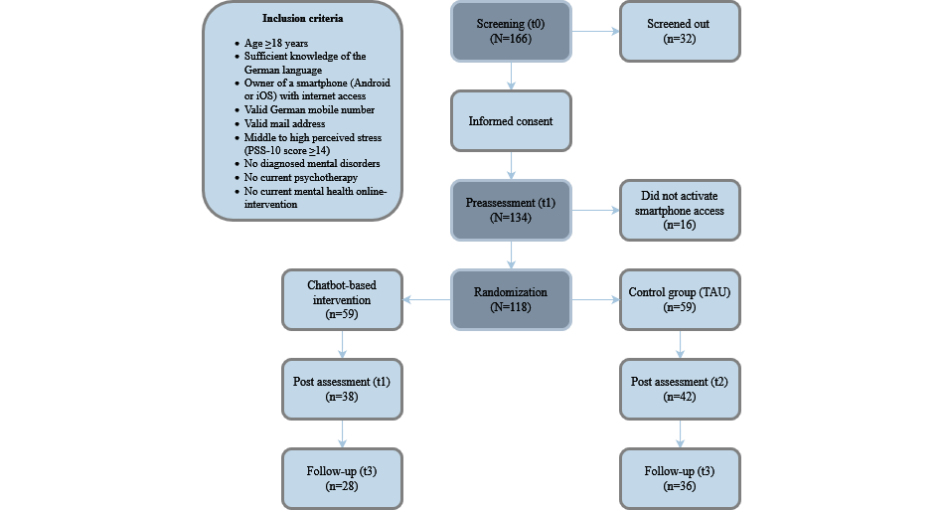
Flowchart of the study procedure. PSS-10: 10-item Perceived Stress Scale; TAU: treatment as usual.

Figure 1 provides a schematic of the study procedure including the final numbers of participants.

### Intervention

**Figure 2:**
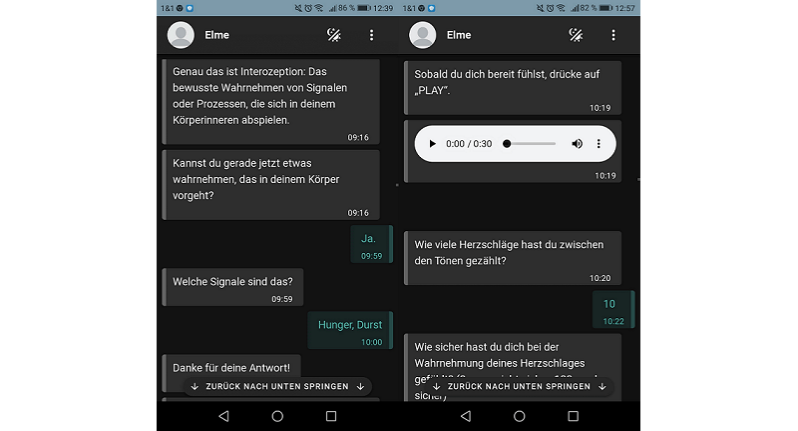
Screenshots of representative dialogues in the interaction between the chatbot and a participant (in German).

ELME is a rule-based chatbot, implemented as a web-based mobile app. ELME offers psychoeducation, exercises in real-time dialogues with the chatbot, audio files, and individual feedback. Sessions were held twice a day (for approximately 10-20 minutes each) over 3 weeks and with flexible timing. Participants could postpone exercises and receive SMS text message reminders. For more detailed intervention information and the detailed procedure, see descriptions in the study protocol [34]. Examples of representative dialogues of the interaction between the chatbot and a participant are depicted in Figure 2.

### Ethical Considerations

All study procedures were approved by the ethics committee of Ulm University (application number 401/20). Written informed consent was obtained from all participants prior to their participation. As an incentive, participants could take part in the intervention for free and received the chance to win a €25 (approximately US $26) gift card from an online shop or, as a student participant, to receive 5 course credits as expense allowance for completing the questionnaires. A further incentive was the possible access to two relaxing exercises and to obtain individual summaries regarding the change in the participants’ health-related parameters from preintervention to follow-up after completing the T3 questionnaire.

### Outcome Assessments

#### Primary Outcome: Perceived Stress

The PSS-10 [36] was used as a screening questionnaire. At T1 to T3, perceived stress was assessed via the 4-item short scale (PSS-4). The ratings on both scales, ranging from 0=“never” to 4=“very often,” were calculated as sum scores, with higher scores representing higher perceived stress.

#### Secondary Outcomes

##### Mindfulness

The 14-item short version of the Freiburg Mindfulness Inventory [37] was used to assess mindfulness. Answers were rated on a 4-point Likert scale ranging from 1=“rarely” to 4=“almost always.” A sum score (range 14-56) was calculated, with higher scores indicating higher mindfulness.

##### Interoceptive Sensibility

Interoceptive sensibility was measured by German versions of the Interoceptive Accuracy Scale (IAS) [38] and the “Awareness” subscale of the Body Perception Questionnaire (BPQ) [39]. The 21-item IAS was rated on a 5-point Likert scale ranging from 1=“strongly disagree” to 5=“strongly agree.” Higher sum scores (range 21-105) indicate greater interoceptive sensibility. The “Awareness” subscale of the BPQ consists of 45 items rated on a 5-point Likert scale ranging from 1=“never” to 5=“always.”

##### Subjective Well-Being

The 5-item WHO Well-Being Index [40,41] was used to assess subjective well-being. Participants responded on a 5-point Likert scale ranging from 5=“all of the time” to 0=“at no time.” A total sum score (range 0-100, with 100 indicating the best well-being) was calculated from raw scores (range 0-25) and multiplied by 4.

##### Emotion Regulation

The German version [42] of the Emotion Regulation Questionnaire [43] was used to assess emotion regulation. The 10-item questionnaire included 6 items representing emotion regulation strategy reappraisal and 4 items assessing emotion regulation strategy suppression, rated on a 7-point Likert scale ranging from 1=“strongly disagree” to 7=“strongly agree.” Accordingly, the mean scores reflect the use of and preferences for various emotion regulation strategies.

##### Ecological Momentary Assessment

Momentary perceived stress and momentary interoceptive sensibility were measured via EMAs twice a day (in the morning and in the afternoon). Momentary perceived stress was assessed via two adapted items for the momentary use of the PSS-4 [36]: “Do you feel that things are going your way?” and “Do you find you can cope with all the things that you have to do?” The items “How present do you feel at the moment?” and “How aware are you of your own body at the moment?” [31,44] were used to measure momentary body awareness. To assess interoceptive sensibility, we used a self-developed question, taking the heartbeat perception task developed by Schandry [45] into account: “How intense do you perceive your heartbeat in the moment?” All rating scales were presented as visual analog scales ranging from 0=“not at all” to 100=“very much.”

##### Mental Health App Usability Questionnaire

To assess the usability of the chatbot as a mental health app, a self-translated German version of the 18-item Mental Health App Usability Questionnaire [46] was used, rated on a scale ranging from 1=“strongly agree” to 7=“strongly disagree.” The questionnaire comprises the following three subscales: ease of use (5 items), interface and satisfaction (7 items), and usefulness (6 items). Mean scores for each subscale were calculated as a total mean score, with lower scores reflecting higher usability.

##### Adherence, Potential Dropout Reasons, and User Feedback

Adherence to the intervention was operationalized by the percentage of completed modules of the intervention. Reasons for potential dropout were assessed via the Dropout Reasons Questionnaire for Internet Interventions [47]. User feedback questions asking the participants if they liked the training (range 1-10) and judging the extent of the training (1="too short" to 12="too long") were also assessed.

### Data Analysis

Data analyses were performed according to the intention-to-treat principle. Due to the nested longitudinal data structure, hierarchical linear regression models were constructed to investigate the intervention effects over time. The measurement points (level 1) were nested within the participants (level 2). The regression analyses include the 3 measurement points preintervention (T1), post intervention (T2), and follow-up (T3). We analyzed hierarchical linear models and model comparisons in R using the packages lme4 [48], lmerTest [49], and r2mlm [50]. The predictor variable time had an interpretable 0 point and the dichotomous predictor group was dummy-coded. Due to assumed interindividual and intraindividual differences in all outcome variables, random-intercept, random-slope models were calculated. The restricted maximum-likelihood estimator was applied for parameter estimation, as it is generally considered to be less biased compared to the maximum-likelihood estimation [51]. We here report the main results that address hypotheses (1) and (2). The significance level for all analyses was set to *P*≤.05.

## Results

### Participant Characteristics

A total sample of 118 participants was randomized to the intervention group (n=59; 72% female) and to the control group (n=59; 81% female). The relevant descriptive statistics at T1 are summarized in Table 1; there were no significant differences between the groups at T1.

**Table 1:** Comparison of relevant participant characteristics at baseline.

Characteristics	Intervention group (n=59), mean (SD)	Control group (n=59), mean (SD)	*t* (*df*=116)	*P* value
Age	33.117 (11.778)	33.085 (13.853)	–0.014	.99
Perceived stress	8.017 (2.701)	7.627 (2.355)	–0.836	.41
Mindfulness	34.271 (5.825)	34.051 (6.957)	0.187	.85
IS^a^ (IAS^b^)	82.220 (10.992)	80.475 (9.233)	–0.934	.35
IS (BPQ^c^)	3.252 (.793)	3.305 (.561)	0.443	.66
Well-being	41.153 (17.341)	42.780 (17.297)	0.510	.61
Emotion regulation: reappraisal	4.130 (1.139)	4.429 (.934)	1.561	.12
Emotion regulation: suppression	3.725 (1.260)	3.339 (1.227)	–1.684	.10

^a^IS: interoceptive sensibility.

^b^IAS: Interoceptive Accuracy Scale.

^c^BPQ: Body Perception Questionnaire.

### Perceived Stress

According to the model regarding perceived stress (Table 2), the nonsignificant fixed effect of the level-1 predictor time indicated that the stress levels did not change over time (from T1 to T3). The fixed effect of the level-2 predictor group and the cross-level interaction of the variables time and group were also not significant. The results of the two models predicting momentary perceived stress showed neither significant main effects of time and group nor their interactions (see Tables S1 and S2 in Multimedia Appendix 1).

**Table 2:** Random-intercept, random-slope model for perceived stress with the predictors time, group, and their interaction.

Effects			Coefficient^a^ (SE or SD)		*df*		*t*		*P* value
**Fixed effects**									
	Intercept	8.135 (.337)		122.119		24.141		<.001	
	Level 1: time	–.341 (.222)		141.471		–1.538		.13	
	Level 2: group	.172 (.478)		123.037		0.359		.72	
	Cross-level interaction (time×group)	–.018 (.329)		140.501		–0.055		.96	
**Random effects (variance components)**									
	*σ*_*u0j*_ (Intercept)	3.243 (1.801)		—^b^		—		—	
	*σ²*_*u01j*_ (Time)	0.017 (0.132)		—		—		—	
	*σ²*_*rij*_ (Residual)	3.916 (1.979)		—		—		—	

^a^Fixed-effects coefficients are β values reported with SEs; random-effects coefficients are *σ²* (variance) values reported with SDs.

^b^Not applicable.

### Mindfulness

The results of the model regarding mindfulness (Table 3) showed no significant fixed effects for time and group. However, the cross-level interaction of time and group was significant.

**Table 3:** Random-intercept, random-slope model for mindfulness with the predictors time, group, and their interaction.

Effects		Coefficient^a^ (SE or SD)	*df*	*t*	*P* value
**Fixed effects**					
	Intercept	34.362 (.826)	115.647	41.589	<.001
	Level 1: time	.078 (.350)	68.640	0.223	.84
	Level 2: group	–.080 (1.170)	116.058	–0.069	.95
	Cross-level-interaction (time×group)	1.130	71.363	2.171	.03
**Random effects (variance components)**					
	*σ²*_*u0j*_ (Intercept)	31.906 (5.649)	—^b^	—	—
	*σ²*_*u01j*_ (Time)	0.113 (0.336)	—	—	—
	*σ²*_*rij*_ (Residual)	9.461 (3.076)	—	—	—

^a^Fixed-effects coefficients are β values reported with SEs; random-effects coefficients are *σ²* (variance) values reported with SDs.

^b^Not applicable.

### Interoceptive Sensibility

The results of the model predicting interoceptive sensibility (assessed via the IAS) revealed neither significant main effects of time or group nor their interaction (see Table S3 in Multimedia Appendix 1). Similarly, assessments via the BPQ showed no significant effects (see Table S4 in Multimedia Appendix 1).

Momentary interoceptive sensibility increased on average over time in both groups. However, the main effect for group and the cross-level interaction of time and group were not significant (see Table S5 in Multimedia Appendix 1).

### Well-Being

As shown in Table 4, subjective well-being improved over time in both groups on average. However, there were neither significant differences in well-being for the groups nor over both time and group.

**Table 4:** Random-intercept, random-slope model for well-being with the predictors time, group, and their interaction.

Effects			Coefficient^a^ (SE or SD)		*df*		*t*		*P* value
**Fixed effects**									
	Intercept	42.150 (2.236)		115.591		18.848		< .001	
	Level 1 (time)	4.237 (1.479)		74.662		2.865		.005	
	Level 2 (group)	–.032 (3.168)		116.363		–0.010		.99	
	Cross-level interaction (time×group)	2.312 (2.189)		78.034		1.056		.29	
**Random effects (variance components)**									
	*σ²*_*u0j*_ (Intercept)	185.17 (13.608)		—^b^		—		—	
	*σ²*_*u01j*_ (Time)	26.580 (5.156)		—		—		—	
	*σ²*_*rij*_ (Residual)	124.180 (11.144)		—		—		—	

^a^Fixed-effects coefficients are β values reported with SEs; random-effects coefficients are *σ²* (variance) values reported with SDs.

^b^Not applicable.

### Emotion Regulation: Reappraisal Subfacet

The results of the model concerning the subfacet reappraisal of emotion regulation (Table 5) revealed neither a significant effect of time nor of group. However, the cross-level interaction of time and group was significant.

**Table 5:** Random-intercept, random-slope model for the emotion regulation reappraisal subfacet with the predictors time, group, and their interaction.

Effects			Coefficient^a^ (SE or SD)		*df*		*t*		*P* value
**Fixed effects**									
	Intercept	4.426 (.133)		116.328		33.297		< .001	
	Level 1: time	.022 (.064)		127.10		0.347		.73	
	Level 2: group	–.255 (.188)		116.819		–1.353		.18	
	Cross-level interaction (time×group)	.223 (.096)		127.085		2.331		.02	
**Random effects (variance components)**									
	*σ²*_*u0j*_ (Intercept)	0.775 (0.880)		—^b^		—		—	
	*σ²*_*u01j*_ (Time)	0.006 (0.075)		—		—		—	
	*σ²*_*rij*_ (Residual)	0.302 (0.550)		—		—		—	

^a^Fixed-effects coefficients are β values reported with SEs; random-effects coefficients are *σ²* (variance) values reported with SDs.

^b^Not applicable.

### Emotion Regulation: Suppression Subfacet

Results regarding the suppression subfacet of emotion regulation revealed no significant changes (see Table S6 in Multimedia Appendix 1).

### Adherence, Dropout Reasons, and User Feedback

The mean adherence (percentage of completed modules) was 58% for the 59 participants in the intervention group; 23 participants skipped intervention units, with "no time" cited as the main reason (n=19). In addition, 22 participants reported technical problems. The average answer rate of the EMA questions was 48% in the intervention group and 66% in the control group. In response to the question if the participants liked the training, the mean score was 6.95 (SD 1.86). The extent of the training was rated a mean score of 7.62.

### Usability

The mean usability score (total score) was 2.55 (SD 0.68), with means of the subscales “ease of use,” “interface and satisfaction,” and “usefulness” of 1.85 (SD 1.01), 2.62 (SD 1.08), and 3.2 (SD 0.94), respectively.

## Discussion

### Principal Results

The aim of this study was to examine the effects of a 3-week chatbot-based intervention on perceived stress and various health-related parameters in stressed individuals. The results show no significant changes in perceived stress after the intervention. There was a significant increase in mindfulness and in emotion regulation as assessed by the subfacet reappraisal in the intervention group over time, whereas there was no change in the suppression subfacet of emotion regulation. Well-being and momentary interoceptive sensibility increased in both groups over time.

### Comparison With Prior Work

#### Effects on Perceived Stress

The nonsignificant reduction in perceived stress is in line with the findings of a similar intervention study [52] and a pilot study [22]; however, considering statistical power problems of these studies, the intervention duration or intensity might be one factor to consider for interpreting the missing effects of the present study. Another explanation could be that there might have been greater initial focus on stress perception, which would potentially buffer the stress-reducing effects due to the intervention. This is supported by findings from psychotherapeutic interventions [53,54], in which the hypothesized effects on psychological outcomes were only detected later because of the confrontation with emotionally charging topics. Furthermore, the results of studies by Baer et al [55] and Venkatesan et al [56] indicated that the effects on perceived stress might become (more) visible after a longer duration of the intervention.

The results regarding momentary perceived stress are in line with previous studies evaluating the effects of 3-month mindfulness-based interventions [57,58]. Moreover, considering the mean adherence of 58% for the present intervention, the mean answer rates of the EMA questions need to be considered when interpreting the results.

#### Effects on Mindfulness

The significant increase in mindfulness is in line with previous findings from online mindfulness-based interventions (eg, [2,3]), indicating that the 3-week chatbot-based intervention comprising mindfulness-based content has the potential to increase mindfulness over time in a stressed sample. A possible mechanism might be that the contents of the intervention addressing mindfulness, stress, and interoception support mindfulness. Nevertheless, mindfulness needs to be interpreted as a secondary outcome in this study.

#### Effects on Interoceptive Sensibility

The missing effects of interoceptive sensibility in this study are in contrast to previous positive effects found for diverse mindfulness-based interventions (eg, [31,32,59]). However, these effects were found in the context of interventions lasting at least 8 weeks. In particular, and in line with the present findings, a 1-week mindfulness-based intervention [32] or a 3-week heartbeat perception training [60] could not improve interoceptive abilities. The findings of this study support the conclusions put forth by Fischer et al [59], Bornemann and Singer [31], and Schillings et al [60] that a longer intervention might be necessary to effectively improve interoceptive abilities. Moreover, previous studies differed in the methods used to assess diverse dimensions of interoceptive abilities (eg, [61,62]). Finally, a longer intervention design of such an innovative chatbot-based intervention might only be reasonable after initial trials with a shorter intervention design such as that of 3 weeks used in this study.

Due to the innovative EMA questions in this study and another study design not including an intervention, the results are not comparable to the previous EMA study by Höller et al [63]. The significant increase in momentary interoceptive sensibility could be explained by a training effect of frequent EMAs, which took place twice a day over 3 weeks.

#### Effects on Emotion Regulation

In line with the results regarding reappraisal, a recent systematic review and meta-analysis on mental health apps to promote emotion regulation and positive mental health in the general population [64] found a medium effect size (*g*=0.49) for emotion regulation compared to control conditions. However, it must be emphasized that this effect was based on only 6 studies, reflecting the lack of RCTs on chatbot-based interventions addressing emotion regulation.

#### Effects on Well-Being

The increase in well-being is in line with comparable previous studies [18,21,22,65] considering differences in the study designs and samples. However, well-being also improved in the control group of this study, which might have also been induced by the daily EMAs as potential positive triggers or observational processes.

### Strengths and Limitations

To the best of our knowledge, this is the first chatbot-based intervention study including contents and assessments on interoception, as well as its association with mindfulness and stress. Further strengths of the study are the highly standardized design in line with the CONSORT (Consolidated Standards of Reporting Trials) guidelines [66,67] and EMAs of interoceptive sensibility [44,57,63]. Furthermore, the design and the usability of the chatbot were successfully tested in a previous feasibility study [35]. Therefore, the chatbot fulfills the required standards of chatbots for mental health support [6]. Finally, the results indicate the high usability of the chatbot.

Limitations of this study should be mentioned and considered for the design of future studies. First, the adherence of the intervention was relatively low at only 58%. Nevertheless, this adherence rate is on average as compared to other online mindfulness-based interventions with adherence rates ranging from 35% to 92% [68]. It should also be noted that adherence rates of digital or chatbot-based interventions were often not reported or operationalized by diverse assessments [20,69] and lack of long-term user engagement in eHealth is a common problem [70,71]. Second, there was a majority of female participants in this study, representing 77% of the sample. Therefore, future intervention studies should consider diverse strategies to specifically address male participants. Third, this study exclusively assessed self-report data. Due to potential differences to objective physiological data [72], future studies should assess both subjective and objective data, especially regarding stress and interoception. Lastly, a text- and rule-based chatbot as used in this study might lack human-like characteristics, such as those regarding the type of interaction between the chatbot and the user. Recent meta-analyses [73,74] showed that chatbot-based studies are more effective when diverse input and output modalities are combined. A multimodal chatbot might be superior because it will appear to be more lively and flexible in dialogues with the user [75] and ready to interact.

### Conclusions and Future Research

To gain insight into how such interventions can be improved to achieve their full potential for stress reduction, besides a longer intervention duration, specific sample groups should be considered, such as employees, diverse age groups, and clinical or subclinical populations, aiming to adapt to individual needs and preferences in everyday life. A chatbot-based intervention seems to have the potential to improve mindfulness and emotion regulation in a stressed sample. Additional factors such as the participants’ social motivation regarding the guidance by the chatbot and the personality of the chatbot [70,76] would be of further interest to foster the alliance or a therapeutic relationship between the user or a patient and the chatbot. Future studies should also investigate the specific elements that have the greatest effects to improve diverse health parameters, such as psychoeducation or exercises. Future research should implement large language models to provide and further develop diverse artificial intelligence (AI) chatbots in digital mental health interventions [77,78]. Recent findings such as those showing that AI-based chatbots are more effective in clinical or subclinical populations [74] need to be considered. Nevertheless, besides the potential of AI-based chatbots for a professional mental health service, emerging reputational risks of AI-based chatbots such as safety and data privacy issues [79,80]; gender, ethnic, and socioeconomic biases [81]; limited empathy and emotional awareness as compared to a human counterpart [82]; and hallucinations [83] should be discussed extensively.

In summary, based on the numerous prospects of chatbots in the psychological and medical field, such as counselling, psychotherapy, diagnostic assessment, and interventions [23,84,85], future studies are needed to derive robust implications in these fields.

## Appendix

Multimedia Appendix 1 Model results for ecological momentary assessments of perceived stress “coping with things” (Table S1) and “feeling on top of things” (Table S2) items. Model results for interoceptive sensibility, including Interoceptive Accuracy Scale (Table S3), Body Perception Questionnaire (Table S4), and momentary assessment (Table S5). Model results for the emotion regulation suppression subfacet (Table S6).

Multimedia Appendix 2 CONSORT-EHEALTH checklist (V 1.6.1).

## Abbreviations

AI: artificial intelligence
BPQ: Body Perception Questionnaire
CONSORT: Consolidated Standards of Reporting Trials
EMA: ecological momentary assessment
IAS: Interoceptive Accuracy Scale
PSS: Perceived Stress Scale
RCT: randomized controlled trial
T0: screening
T1: preintervention
T2: post intervention (3 weeks)
T3: follow-up (6 weeks)
WHO: World Health Organization

## Data Availability

The data sets generated during and/or analyzed during this study are available from the corresponding author on reasonable request.

## References

[1] Salari N, Hosseinian-Far A, Jalali R, Vaisi-Raygani A, Rasoulpoor S, Mohammadi M, Rasoulpoor S, Khaledi-Paveh B (2020). Prevalence of stress, anxiety, depression among the general population during the COVID-19 pandemic: a systematic review and meta-analysis. Global Health.

[2] Zhang Y, Xue J, Huang Y (2020). A meta-analysis: internet mindfulness-based interventions for stress management in the general population. Medicine.

[3] Spijkerman MPJ, Pots WTM, Bohlmeijer ET (2016). Effectiveness of online mindfulness-based interventions in improving mental health: a review and meta-analysis of randomised controlled trials. Clin Psychol Rev.

[4] Nguyen-Feng VN, Greer CS, Frazier P (2017). Using online interventions to deliver college student mental health resources: evidence from randomized clinical trials. Psychol Serv.

[5] Ma Y, She Z, Siu AF, Zeng X, Liu X (2018). Effectiveness of online mindfulness-based interventions on psychological distress and the mediating role of emotion regulation. Front Psychol.

[6] Kretzschmar K, Tyroll H, Pavarini G, Manzini A, Singh I, NeurOx Young People’s Advisory Group (2019). Can your phone be your therapist? Young people's ethical perspectives on the use of fully automated conversational agents (chatbots) in mental health support. Biomed Inform Insights.

[7] Musiat P, Johnson C, Atkinson M, Wilksch S, Wade T (2022). Impact of guidance on intervention adherence in computerised interventions for mental health problems: a meta-analysis. Psychol Med.

[8] Domhardt M, Geßlein H, von Rezori RE, Baumeister H (2019). Internet- and mobile-based interventions for anxiety disorders: a meta-analytic review of intervention components. Depress Anxiety.

[9] Ebert DD, Buntrock C, Lehr D, Smit F, Riper H, Baumeister H, Cuijpers P, Berking M (2018). Effectiveness of web- and mobile-based treatment of subthreshold depression with adherence-focused guidance: a single-blind randomized controlled trial. Behav Ther.

[10] Adamopoulou E, Moussiades L (2020). Chatbots: history, technology, and applications. Machine Learn Applic.

[11] Brandtzaeg P, Følstad A (2017). Why people use chatbots.

[12] Bendig E, Erb B, Schulze-Thuesing L, Baumeister H (2019). Die nächste generation: chatbots in der klinischen psychologie und psychotherapie zur förderung mentaler gesundheit – ein scoping-review. Verhaltenstherapie.

[13] Hill J, Randolph Ford W, Farreras IG (2015). Real conversations with artificial intelligence: a comparison between human–human online conversations and human–chatbot conversations. Comput Hum Behav.

[14] Müschenich M, Wamprecht L (2018). Wie gehts uns denn morgen? [Health 4.0 - how are we doing tomorrow?]. Bundesgesundheitsblatt Gesundheitsforschung Gesundheitsschutz.

[15] Gamble A (2020). Artificial intelligence and mobile apps for mental healthcare: a social informatics perspective. Aslib J Inf Manag.

[16] Stieger M, Nißen M, Rüegger D, Kowatsch T, Flückiger C, Allemand M (2018). PEACH, a smartphone- and conversational agent-based coaching intervention for intentional personality change: study protocol of a randomized, wait-list controlled trial. BMC Psychol.

[17] Vaidyam AN, Wisniewski H, Halamka JD, Kashavan MS, Torous JB (2019). Chatbots and conversational agents in mental health: a review of the psychiatric landscape. Can J Psychiatry.

[18] Suganuma S, Sakamoto D, Shimoyama H (2018). An embodied conversational agent for unguided internet-based cognitive behavior therapy in preventative mental health: feasibility and acceptability pilot trial. JMIR Ment Health.

[19] Gaffney H, Mansell W, Tai S (2019). Conversational agents in the treatment of mental health problems: mixed-method systematic review. JMIR Ment Health.

[20] Vaidyam AN, Linggonegoro D, Torous J (2021). Changes to the psychiatric chatbot landscape: a systematic review of conversational agents in serious mental illness. Can J Psychiatry.

[21] Potts C, Lindström F, Bond R, Mulvenna M, Booth F, Ennis E, Parding K, Kostenius C, Broderick T, Boyd K, Vartiainen A, Nieminen H, Burns C, Bickerdike A, Kuosmanen L, Dhanapala I, Vakaloudis A, Cahill B, MacInnes M, Malcolm M, O'Neill S (2023). A multilingual digital mental health and well-being chatbot (ChatPal): pre-post multicenter intervention study. J Med Internet Res.

[22] Ly KH, Ly A, Andersson G (2017). A fully automated conversational agent for promoting mental well-being: a pilot RCT using mixed methods. Internet Interv.

[23] Bendig E, Erb B, Schulze-Thuesing L, Baumeister H (2019). The next generation: chatbots in clinical psychology and psychotherapy to foster mental health – a scoping review. Verhaltenstherapie.

[24] Laranjo L, Dunn AG, Tong HL, Kocaballi AB, Chen J, Bashir R, Surian D, Gallego B, Magrabi F, Lau AYS, Coiera E (2018). Conversational agents in healthcare: a systematic review. J Am Med Inform Assoc.

[25] Abd-Alrazaq AA, Alajlani M, Alalwan AA, Bewick BM, Gardner P, Househ M (2019). An overview of the features of chatbots in mental health: a scoping review. Int J Med Inform.

[26] Khalsa SS, Adolphs R, Cameron OG, Critchley HD, Davenport PW, Feinstein JS, Feusner JD, Garfinkel SN, Lane RD, Mehling WE, Meuret AE, Nemeroff CB, Oppenheimer S, Petzschner FH, Pollatos O, Rhudy JL, Schramm LP, Simmons WK, Stein MB, Stephan KE, Van den Bergh O, Van Diest I, von Leupoldt A, Paulus MP, Interoception Summit 2016 participants (2018). Interoception and mental health: a roadmap. Biol Psychiatry Cogn Neurosci Neuroimaging.

[27] Craig AD (2002). How do you feel? Interoception: the sense of the physiological condition of the body. Nat Rev Neurosci.

[28] Fischer D, Berberich G, Zaudig M, Krauseneck T, Weiss S, Pollatos O (2016). Interoceptive processes in anorexia nervosa in the time course of cognitive-behavioral therapy: a pilot study. Front Psychiatry.

[29] Eggart M, Lange A, Binser MJ, Queri S, Müller-Oerlinghausen B (2019). Major depressive disorder is associated with impaired interoceptive accuracy: a systematic review. Brain Sci.

[30] Schultchen D, Bayer J, Kühnel J, Melchers KG, Pollatos O (2019). Interoceptive accuracy is related to long-term stress via self-regulation. Psychophysiology.

[31] Bornemann B, Singer T (2017). Taking time to feel our body: steady increases in heartbeat perception accuracy and decreases in alexithymia over 9 months of contemplative mental training. Psychophysiology.

[32] Parkin L, Morgan R, Rosselli A, Howard M, Sheppard A, Evans D, Hawkins A, Martinelli M, Golden A, Dalgleish T, Dunn B (2013). Exploring the relationship between mindfulness and cardiac perception. Mindfulness.

[33] Balaskas A, Schueller SM, Cox AL, Doherty G (2021). Ecological momentary interventions for mental health: a scoping review. PLoS One.

[34] Schillings C, Meissner D, Erb B, Schultchen D, Bendig E, Pollatos O (2023). A chatbot-based intervention with ELME to improve stress and health-related parameters in a stressed sample: study protocol of a randomised controlled trial. Front Digit Health.

[35] Feasibility study of a chatbot-based training for stress reduction and health improvement. German Clinical Trials Register.

[36] Cohen S, Kamarck T, Mermelstein R (1983). A global measure of perceived stress. J Health Soc Behav.

[37] Walach H, Buchheld N, Buttenmüller V, Kleinknecht N, Schmidt S (2006). Measuring mindfulness—the Freiburg Mindfulness Inventory (FMI). Person Individ Diff.

[38] Murphy J, Brewer R, Plans D, Khalsa SS, Catmur C, Bird G (2020). Testing the independence of self-reported interoceptive accuracy and attention. Q J Exp Psychol.

[39] The Body Perception Questionnaire. Traumatic Stress Research Consortium.

[40] World Health Organization (1998). WHO Info Package: Mastering depression in Primary Care.

[41] Topp CW, Østergaard SD, Søndergaard S, Bech P (2015). The WHO-5 Well-Being Index: a systematic review of the literature. Psychother Psychosom.

[42] Abler B, Kessler H (2009). Emotion Regulation Questionnaire – Eine deutschsprachige Fassung des ERQ von Gross und John. Diagnostica.

[43] Gross JJ, John OP (2003). Individual differences in two emotion regulation processes: implications for affect, relationships, and well-being. J Pers Soc Psychol.

[44] Singer T, Kok B, Bornemann B, Zurborg S, Bolz M, Bochow C (2016). The ReSource Project: Background, Design, Samples, and Measurements. Second edition.

[45] Schandry R (1981). Heart beat perception and emotional experience. Psychophysiology.

[46] Zhou L, Bao J, Setiawan IMA, Saptono A, Parmanto B (2019). The mHealth App Usability Questionnaire (MAUQ): development and validation study. JMIR Mhealth Uhealth.

[47] Bendig E, Braun L, Simon L (2020). Dropout Reasons Questionnaire for Internet Interventions (DRQi) - Questionnaire for the Systematic Recording of Dropout Reasons at Different Stages in the Implementation of an Online Intervention.

[48] Bates D, Mächler M, Bolker B, Walker S (2015). Fitting linear mixed-effects models using lme4. J Stat Soft.

[49] Kuznetsova A, Brockhoff PB, Christensen RHB (2017). lmerTest package: tests in linear mixed effects models. J Stat Soft.

[50] Rights JD, Sterba SK (2019). Quantifying explained variance in multilevel models: an integrative framework for defining R-squared measures. Psychol Methods.

[51] Finch W, Bolin J, Kelley K (2019). Multilevel Modeling Using R. Second edition.

[52] Gardiner PM, McCue KD, Negash LM, Cheng T, White LF, Yinusa-Nyahkoon L, Jack BW, Bickmore TW (2017). Engaging women with an embodied conversational agent to deliver mindfulness and lifestyle recommendations: a feasibility randomized control trial. Patient Educ Couns.

[53] Schauenburg H, Sammet I, Strack M (2001). Course of symptom severity and prediction of outcome in inpatient psychotherapy. Z Psychosom Med Psychother.

[54] Owen J, Adelson J, Budge S, Wampold B, Kopta M, Minami T, Miller S (2015). Trajectories of change in psychotherapy. J Clin Psychol.

[55] Baer RA, Carmody J, Hunsinger M (2012). Weekly change in mindfulness and perceived stress in a mindfulness-based stress reduction program. J Clin Psychol.

[56] Venkatesan A, Krymis H, Scharff J, Waber A (2021). Changes in perceived stress following a 10-week digital mindfulness-based stress reduction program: retrospective study. JMIR Form Res.

[57] Linz R, Puhlmann LMC, Engert V, Singer T (2022). Investigating the impact of distinct contemplative mental trainings on daily life stress, thoughts and affect-Evidence from a nine-month longitudinal ecological momentary assessment study. Psychoneuroendocrinology.

[58] Aguilar-Raab C, Stoffel M, Hernández C, Rahn S, Moessner M, Steinhilber B, Ditzen B (2021). Effects of a mindfulness-based intervention on mindfulness, stress, salivary alpha-amylase and cortisol in everyday life. Psychophysiology.

[59] Fischer D, Messner M, Pollatos O (2017). Improvement of interoceptive processes after an 8-week body scan intervention. Front Hum Neurosci.

[60] Schillings C, Karanassios G, Schulte N, Schultchen D, Pollatos O (2022). The effects of a 3-week heartbeat perception training on interoceptive abilities. Front Neurosci.

[61] Garfinkel SN, Seth AK, Barrett AB, Suzuki K, Critchley HD (2015). Knowing your own heart: distinguishing interoceptive accuracy from interoceptive awareness. Biol Psychol.

[62] Murphy J, Catmur C, Bird G (2019). Classifying individual differences in interoception: implications for the measurement of interoceptive awareness. Psychon Bull Rev.

[63] Höller I, Stenzel J, Rath D, Forkmann T (2021). Listen to your heart-ecological momentary assessment of interoceptive accuracy, awareness and sensibility: a pilot study. Int J Environ Res Public Health.

[64] Eisenstadt M, Liverpool S, Infanti E, Ciuvat RM, Carlsson C (2021). Mobile apps that promote emotion regulation, positive mental health, and well-being in the general population: systematic review and meta-analysis. JMIR Ment Health.

[65] Bendig E, Erb B, Meißner D, Bauereiß N, Baumeister H (2021). Feasibility of a Software agent providing a brief Intervention for Self-help to Uplift psychological wellbeing ("SISU"). A single-group pretest-posttest trial investigating the potential of SISU to act as therapeutic agent. Internet Interv.

[66] Eysenbach G, CONSORT-EHEALTH Group (2011). CONSORT-EHEALTH: improving and standardizing evaluation reports of web-based and mobile health interventions. J Med Internet Res.

[67] Schulz KF, Altman DG, Moher D, CONSORT Group (2010). CONSORT 2010 statement: updated guidelines for reporting parallel group randomised trials. BMJ.

[68] Sommers-Spijkerman M, Austin J, Bohlmeijer E, Pots W (2021). New evidence in the booming field of online mindfulness: an updated meta-analysis of randomized controlled trials. JMIR Ment Health.

[69] Beintner I, Vollert B, Zarski A, Bolinski F, Musiat P, Görlich D, Ebert DD, Jacobi C (2019). Adherence reporting in randomized controlled trials examining manualized multisession online interventions: systematic review of practices and proposal for reporting standards. J Med Internet Res.

[70] Koulouri T, Macredie RD, Olakitan D (2022). Chatbots to support young adults’ mental health: an exploratory study of acceptability. ACM Trans Interact Intell Syst.

[71] Torous J, Nicholas J, Larsen ME, Firth J, Christensen H (2018). Clinical review of user engagement with mental health smartphone apps: evidence, theory and improvements. Evid Based Ment Health.

[72] Zuniga Gonzalez DA, Richards D, Bilgin AA (2021). Making it real: a study of augmented virtuality on presence and enhanced benefits of study stress reduction sessions. Int J Hum Comput Stud.

[73] Lim SM, Shiau CWC, Cheng LJ, Lau Y (2022). Chatbot-delivered psychotherapy for adults with depressive and anxiety symptoms: a systematic review and meta-regression. Behav Ther.

[74] Li H, Zhang R, Lee Y, Kraut RE, Mohr DC (2023). Systematic review and meta-analysis of AI-based conversational agents for promoting mental health and well-being. NPJ Digit Med.

[75] Montenegro JLZ, da Costa CA, da Rosa Righi R (2019). Survey of conversational agents in health. Exp Syst Appl.

[76] Grové C (2020). Co-developing a mental health and wellbeing chatbot with and for young people. Front Psychiatry.

[77] Thakur S, Rastogi D, Singh L (2021). MOODY: a natural language processing-based chatbot for mental health care.

[78] Boucher EM, Harake NR, Ward HE, Stoeckl SE, Vargas J, Minkel J, Parks AC, Zilca R (2021). Artificially intelligent chatbots in digital mental health interventions: a review. Expert Rev Med Devices.

[79] Li J (2023). Security implications of AI chatbots in health care. J Med Internet Res.

[80] De Freitas J, Uğuralp AK, Oğuz‐Uğuralp Z, Puntoni S (2023). Chatbots and mental health: insights into the safety of generative AI. J Consum Psychol.

[81] Kim J, Cai ZR, Chen ML, Simard JF, Linos E (2023). Assessing biases in medical decisions via clinician and AI chatbot responses to patient vignettes. JAMA Netw Open.

[82] Pham KT, Nabizadeh A, Selek S (2022). Artificial intelligence and chatbots in psychiatry. Psychiatr Q.

[83] Giuffrè M, You K, Shung DL (2024). Evaluating ChatGPT in medical contexts: the imperative to guard against hallucinations and partial accuracies. Clin Gastroenterol Hepatol.

[84] Pryss R, Kraft R, Baumeister H, Winkler J, Probst T, Reichert M, Langguth B, Spiliopoulou M, Schlee W, Montag C, Baumeister H (2019). Using chatbots to support medical and psychological treatment procedures: challenges, opportunities, technologies, reference architecture. Digital Phenotyping and Mobile Sensing.

[85] Aggarwal A, Tam CC, Wu D, Li X, Qiao S (2023). Artificial intelligence-based chatbots for promoting health behavioral changes: systematic review. J Med Internet Res.

