# Supplementary material for: Effects of a Chatbot-Based Intervention on Stress and Health-Related Parameters in a Stressed Sample: Randomized Controlled Trial
Source: JMIR Ment Health. 2024 May 28;11:e50454. doi: 10.2196/50454 (PMC11167325; doi:10.2196/50454)
Supplement: Multimedia Appendix 1 [file mental_v11i1e50454_app1.docx]

**Multimedia Appendix 1**

***Momentary perceived stress by ecological momentary assessment, item “coping with things”***

Table S1. Random-intercept and random-slope model for perceived stress (item “coping with things”) as assessed via ecological momentary assessment with the predictors time, group, and their interaction.

|  |  |  |  |  | Model |  |  |
| --- | --- | --- | --- | --- | --- | --- | --- |
|  |  |  | *β* | *SE* | *df* | *t* | *p* |
| Fixed effects |  |  |  |  |  |  |  |
|  | Intercept |  | 50.592 | 2.095 | 104.260 | 24.148 | < .001*** |
|  | Level 1 |  |  |  |  |  |  |
|  |  | Time | .033 | .065 | 82.059 | .512 | .610 |
|  | Level 2 |  |  |  |  |  |  |
|  |  | Group | 2.696 | 3.051 | 111.176 | .883 | .379 |
|  |  |  |  |  |  |  |  |
|  | Cross-level-interaction |  |  |  |  |  |  |
|  |  | Time* group | .024 | .010 | 92.408 | .240 | .811 |
|  |  |  |  |  |  |  |  |
|  |  |  | *σ²* | *SD* |  | |  |
| Random effects (Variance components) |  |  |  |  |  |  |  |
|  | *σ²_u0j_*  (Intercept) |  | 211.530 | 14.544 |  |  |  |
|  | *σ²_u01j_* (Time) |  | .124 | .352 |  |  |  |
|  | *σ²_rij_* (Residual) |  | 244.343 | 15.632 |  |  |  |

*Note. β* = fixed effect coefficients; *σ² =* variance of random effect coefficients; *SE* = standard errors; *SD* = standard deviations.

***Momentary perceived stress by ecological momentary assessment, item “feeling on top of things”***

Table S2. Random-intercept and random-slope model for perceived stress (item “feeling on top of things”) as assessed via ecological momentary assessment for the predictors time, group, and their interaction.

|  |  |  |  |  | Model |  |  |
| --- | --- | --- | --- | --- | --- | --- | --- |
|  |  |  | *β* | *SE* | *df* | *t* | *p* |
| Fixed effects |  |  |  |  |  |  |  |
|  | Intercept |  | 52.364 | 2.307 | 102.647 | 22.699 | < .001*** |
|  | Level 1 |  |  |  |  |  |  |
|  |  | Time | -.018 | .076 | 83.131 | -.240 | .811 |
|  | Level 2 |  |  |  |  |  |  |
|  |  | Group | 2.047 | 3.348 | 108.395 | .061 | .542 |
|  |  |  |  |  |  |  |  |
|  | Cross-level-interaction |  |  |  |  |  |  |
|  |  | Time* group | .084 | .115 | 90.645 | .727 | .469 |
|  |  |  |  |  |  |  |  |
|  |  |  | *σ²* | *SD* |  | |  |
| Random effects (Variance components) |  |  |  |  |  |  |  |
|  | *σ²_u0j_*  (Intercept) |  | 267.685 | 16.361 |  |  |  |
|  | *σ²_u01j_* (Time) |  | .211 | .459 |  |  |  |
|  | *σ²_rij_* (Residual) |  | 220.839 | 14.861 |  |  |  |

*Note. β* = fixed effect coefficients; *σ² =* variance of random effect coefficients; *SE* = standard errors; *SD* = standard deviations.

***Interoceptive sensibility (Interoceptive Accuracy Scale)***

Table S3. Random-intercept and random-slope model for interoceptive sensibility as assessed via the Interoceptive Accuracy Scale with the predictors time, group, and their interaction.

|  |  |  |  |  | Model |  |  |
| --- | --- | --- | --- | --- | --- | --- | --- |
|  |  |  | *β* | *SE* | *df* | *t* | *p* |
| Fixed effects |  |  |  |  |  |  |  |
|  | Intercept |  | 80.829 | 1.256 | 115.902 | 64.381 | < .001*** |
|  | Level 1 |  |  |  |  |  |  |
|  |  | Time | .268 | .692 | 68.722 | .387 | .700 |
|  | Level 2 |  |  |  |  |  |  |
|  |  | Group | 1.555 | 1.778 | 116.575 | .875 | .384 |
|  |  |  |  |  |  |  |  |
|  | Cross-level-interaction |  |  |  |  |  |  |
|  |  | Time* group | -.197 | 1.026 | 71.524 | -.192 | .849 |
|  |  |  |  |  |  |  |  |
|  |  |  | *σ²* | *SD* |  | |  |
| Random effects (Variance components) |  |  |  |  |  |  |  |
|  | *σ²_u0j_*  (Intercept) |  | 61.135 | 7.819 |  |  |  |
|  | *σ²_u01j_* (Time) |  | 1.252 | 1.119 |  |  |  |
|  | *σ²_rij_* (Residual) |  | 36.045 | 6.004 |  |  |  |

*Note. β* = fixed effect coefficients; *σ² =* variance of random effect coefficients; *SE* = standard errors; *SD* = standard deviations.

***Interoceptive sensibility (Body Perception Questionnaire)***

Table S4. Random-intercept and random-slope model for interoceptive sensibility as assessed via the Body Perception Questionnaire with the predictors time, group, and their interaction.

|  |  |  |  |  | Model |  |  |
| --- | --- | --- | --- | --- | --- | --- | --- |
|  |  |  | *β* | *SE* | *df* | *t* | *p* |
| Fixed effects |  |  |  |  |  |  |  |
|  | Intercept |  | 3.332 | .081 | 115.571 | 40.974 | < .001*** |
|  | Level 1 |  |  |  |  |  |  |
|  |  | Time | .064 | .052 | 61.720 | 1.220 | .227 |
|  | Level 2 |  |  |  |  |  |  |
|  |  | Group | -.057 | .115 | 116.086 | -.493 | .623 |
|  |  |  |  |  |  |  |  |
|  | Cross-level-interaction |  |  |  |  |  |  |
|  |  | Time* group | .012 | .077 | 64.980 | .156 | .877 |
|  |  |  |  |  |  |  |  |
|  |  |  | *σ²* | *SD* |  | |  |
| Random effects (Variance components) |  |  |  |  |  |  |  |
|  | *σ²_u0j_*  (Intercept) |  | .302 | .549 |  |  |  |
|  | *σ²_u01j_* (Time) |  | .056 | .237 |  |  |  |
|  | *σ²_rij_* (Residual) |  | .099 | .315 |  |  |  |

*Note. β* = fixed effect coefficients; *σ² =* variance of random effect coefficients; *SE* = standard errors; *SD* = standard deviations.

***Momentary interoceptive sensibility***

Table S5. Random-intercept and random-slope model for momentary interoceptive sensibility, with the predictors time, group, and their interaction.

|  |  |  |  |  | Model |  |  |
| --- | --- | --- | --- | --- | --- | --- | --- |
|  |  |  | *β* | *SE* | *df* | *t* | *p* |
| Fixed effects |  |  |  |  |  |  |  |
|  | Intercept |  | 37.901 | 2.477 | 98.989 | 15.300 | < .001*** |
|  | Level 1 |  |  |  |  |  |  |
|  |  | Time | .229 | .076 | 81.461 | 3.018 | .003** |
|  | Level 2 |  |  |  |  |  |  |
|  |  | Group | 4.551 | 3.609 | 104.110 | 1.261 | .210 |
|  |  |  |  |  |  |  |  |
|  | Cross-level-interaction |  |  |  |  |  |  |
|  |  | Time* group | -.137 | .115 | 90.030 | -1.189 | .236 |
|  |  |  |  |  |  |  |  |
|  |  |  | *σ²* | *SD* |  | |  |
| Random effects (Variance components) |  |  |  |  |  |  |  |
|  | *σ²_u0j_*  (Intercept) |  | 284.540 | 16.868 |  |  |  |
|  | *σ²_u01j_* (Time) |  | .158 | .397 |  |  |  |
|  | *σ²_rij_* (Residual) |  | 330.894 | 18.191 |  |  |  |

*Note. β* = fixed effect coefficients; *σ² =* variance of random effect coefficients; *SE* = standard errors; *SD* = standard deviations.

***Emotion regulation subfacet suppression***

Table S6. Random-intercept and random-slope model for the emotion regulation subfacet suppression, with the predictors time, group, and their interaction.

|  |  |  |  |  | Model |  |  |
| --- | --- | --- | --- | --- | --- | --- | --- |
|  |  |  | *β* | *SE* | *df* | *t* | *p* |
| Fixed effects |  |  |  |  |  |  |  |
|  | Intercept |  | 3.326 | .157 | 114.392 | 21.239 | < .001*** |
|  | Level 1 |  |  |  |  |  |  |
|  |  | Time | -.014 | .090 | 72.059 | -.150 | .881 |
|  | Level 2 |  |  |  |  |  |  |
|  |  | Group | .404 | .222 | 114.947 | 1.820 | .071 |
|  |  |  |  |  |  |  |  |
|  | Cross-level-interaction |  |  |  |  |  |  |
|  |  | Time* group | -.151 | .133 | 75.692 | -1.133 | .261 |
|  |  |  |  |  |  |  |  |
|  |  |  | *σ²* | *SD* |  | |  |
| Random effects (Variance components) |  |  |  |  |  |  |  |
|  | *σ²_u0j_*  (Intercept) |  | 1.070 | 1.034 |  |  |  |
|  | *σ²_u01j_* (Time) |  | .112 | .334 |  |  |  |
|  | *σ²_rij_* (Residual) |  | .425 | .652 |  |  |  |

*Note. β* = fixed effect coefficients; *σ² =* variance of random effect coefficients; *SE* = standard errors; *SD* = standard deviations.
